# How the Latest Guidelines Are Changing the Diagnostic and Therapeutic Landscape of Arterial Hypertension

**DOI:** 10.3390/jcm14217694

**Published:** 2025-10-29

**Authors:** Maria Concetta Pastore, Clarissa Carmona De Azevedo Bellagamba, Riccardo Liga, Andrea Stefanini, Miriam Durante, Flavio D’Ascenzi, Roberto Pedrinelli, Matteo Cameli

**Affiliations:** 1 Department of Medical Biotechnologies, Division of Cardiology, University of Siena, 53100 Siena, Italy; clarissabellagamba@gmail.com (C.C.D.A.B.); andreastefanini@gmail.com (A.S.); matteo.cameli@unisi.it (M.C.); 2 Cardiac, Thoracic and Vascular Department, University of Pisa, 56126 Pisa, Italy; riccardo.liga@gmail.com (R.L.); roberto.pedrinelli@unipi.it (R.P.); 3 Dipartimento di Scienze della Vita, University of Siena, Via Aldo Moro 2, 53100 Siena, Italy; miridurante@gmail.it; 4 Sports Cardiology and Rehab Unit, Department of Medical Biotechnologies, University of Siena, 53100 Siena, Italy; flaviodascenzi@unisi.it

**Keywords:** arterial hypertension, guidelines, screening, blood pressure, cardiovascular disease

## Abstract

Arterial hypertension (HTN) represents the major cardiovascular risk factor and still represents a global health issue. In the last two years, three international societies published the new guidelines for the treatment of HTN (European Society of Cardiology, 2025; European Society of Hypertension, 2023; and American College of Cardiology in collaboration with American Heart Association, 2025), with many novelties which have been the object of many discussions among experts. The increased stress on screening programs, the new lower targets to reach by medical treatment, and the new strategies for resistant hypertension have raised some doubt on the feasibility of these recommendations in clinical practice. In this perspective document, the authors highlight and discuss the novelties and potential pros and cons of the new international recommendations for the treatment of arterial HTN.

## 1. Introduction

Cardiovascular diseases (CVDs) are an increasing global health concern as they are the leading cause of death worldwide and hold a significant impact in quality of life. According to the World Health Organization, the global prevalence of hypertension, a major modifiable risk factor for CVDs, is 33% among adults aged 30–79 years, with 44% of this population unaware of this condition. Two-thirds of the hypertensive population live in low- and middle-income countries. In European countries, the prevalence is similar to the overall global prevalence, with between-country differences and values lower than average in some Western and above average in Eastern European countries [1]; among adults in the United States, the prevalence of hypertension is up to 46.7%, with the prevalence varying by age, sex, and race/ethnicity [2], and the overall prevalence of hypertension in the Asia–Pacific region ranges between 10.6% and 48.3% [3]. As hypertension (HTN) represents a major modifiable risk factor for CVDs, such as coronary artery disease, heart failure, stroke, and chronic kidney disease (CKD), the latest clinical practice guidelines on hypertension brought some changes based on evidence that focus on fatal and non-fatal outcomes rather than surrogate outcomes such as blood pressure (BP) lowering alone. These novelties regard the screening procedures, diagnostic methods and criteria, prevention and treatment of elevated blood pressure, and considerations on special populations. This paper will focus on the clinical impact of the new recommendations from the European Society of Cardiology (ESC) Guidelines for the Management of Elevated Blood Pressure and Hypertension published in 2024 [4], the European Society of Hypertension (ESH) Guidelines for the Management of Arterial Hypertension published in 2023 [1], and the American College of Cardiology/American Heart Association (ACC/AHA) 2025 High Blood Pressure Guidelines [2].

Even though a considerable constraint of this review may be the exclusion of guidelines from other continents, such as the Asian region, it is mainly linked to the absence of an official, unified guideline from a single continental society. Given this limitation, we focused on highlighting main recommendations derived from regional consensus documents. The aim of this perspective document is to highlight and discuss the novelties and potential pros and cons of the new international recommendations for the treatment of arterial HTN.

The main revised recommendations of the ESC guidelines and the ACC/AHA guidelines are summarized in Table 1.

## 2. Diagnosis: From Measurement to Additional Examination

As hypertension is mainly an asymptomatic condition that is frequently detected by systemic or opportunistic screening, the guidelines reinforce the importance of those strategies and define the adequate timing for screening for hypertension that should be performed at least every 3 years for adults aged < 40 years and at least annually for adults aged ≥ 40 years and individuals with elevated BP who do not currently meet the risk threshold for BP-lowering treatment [4].

The types of screening can vary and should not necessarily be performed in clinical settings, so it can be feasible in different countries and healthcare systems; in this light, self-screening and non-physician screening are also increasingly used.

Out-of-office measurement of BP is now pivotal, not optional. Both the 2023 ESH and 2024 ESC guidelines agree that home BP measurement (HBPM)/ambulatory BP measurement (ABPM) must be performed to corroborate office readings, detect white-coat/masked HTN, and guide titration in patients already receiving BP-lowering medications. The recently published 2025 ACC/AHA guidelines (2025 High Blood Pressure Guideline), as the previous guidelines of 2017 [2,5], also specifically recommend out-of-office BP measurement for this purpose.

On the other hand, several recommendations were added to standardize office BP measurement, as BP can be influenced by circumstances including position, ambient temperature, the technique of measurement, accuracy of equipment, and physical condition of the patient. However, some protocols are more time-consuming and can be hard to apply in the practical clinical setting according to the time available for each visit, or may delay the diagnosis and treatment, as some guidelines recommend multiple readings on at least two separate occasions to confirm a diagnosis [6,7]. Moreover, the recent stronger emphasis on out-of-office BP measurement for diagnostic purposes may reflect data demonstrating that out-of-office BP measurement is generally more accurate than office BP measurement, better predicts CVD events, and correlates better with left ventricular mass [8].

After the initial diagnosis of hypertension, it is important to pursue a complete clinical evaluation to identify factors potentially contributing to hypertension, identify other CVD risk factors, establish whether there is evidence of hypertension-mediated organ damage (HMOD) or existing cardiac, cerebrovascular or renal disease and, when indicated, screen for potential secondary causes of hypertension (e.g., primary aldosteronism, obstructive sleep apnoea syndrome, renovascular hypertension, renoparenchymal hypertension, phaeochromocytoma/paraganglioma and others).

After diagnosis, routine tests are recommended in the initial work-up to improve the risk stratification, including fasting blood glucose, serum lipids, blood sodium and potassium, hemoglobin and/or hematocrit, calcium and TSH, blood creatinine and estimated glomerular filtration rate (eGFR), and urinalysis and urinary albumin-to-creatinine ratio. A 12-lead ECG is also recommended in the initial work-up, and, in some cases when it is likely to change patient management, other optional tests such as echocardiogram, coronary artery calcium score by cardiac CT, carotid or femoral plaque identification by ultrasound evaluation, high-sensitivity cardiac troponin and/or NT-proBNP, and fundoscopy should be considered to up-classify the risk of these patients if testing is abnormal. The rationale of those recommendations is beyond the sole objective of stratifying the CVD risk and identifying HMOD, as some trials demonstrated that visualizing HMOD may help optimize treatment by facilitating patient adherence and overcoming physician inertia in achieving an intensive BP treatment goal [9] and assuming a fundamental role for the evaluation of patients with arterial HTN.

Moreover, all these exams contribute to the estimation of cardiovascular risk, which should guide medical treatment and setting of therapeutic targets. For those without a previous CV event and/or high cardiovascular risk condition such as established clinical cardiovascular disease, moderate or severe CKD, diabetes mellitus, or familial hypercholesterolaemia, the CVD risk evaluation is recommended to be performed by using the Systematic Coronary Risk Evaluation-2 (SCORE2) and SCORE2-Older Persons (SCORE2-OP) to predict 10-year risk CVD events [4]. The 2025 ACC/AHA guidelines recommend the use PREVENT calculator (Predicting Risk of CVD EVENTs) to identify patients at increased 10-year CVD risk (i.e., ≥7.5% based on PREVENT) [2]. It is important, however, to take in mind that the PREVENT and SCORE2 and SCORE2-OP algorithms cannot be used interchangeably in absence of reliable external validation studies.

## 3. New Targets: Are They Feasible to Reach?

The classification of hypertension has evolved over the years from a simple dichotomous classification (hypertension/not hypertension) to a more nuanced system. Indeed, the main innovation of the 2024 ESC guidelines is the introduction of a simplified categorization of adults according to their BP: non-elevated BP (<120/70 mmHg), elevated BP (120–139/70–89 mmHg), and hypertension (>140/90 mmHg), with impact on treatment threshold according to the individual CVD risk, since using BP thresholds for hypertension alone for allocating treatment would lead to undertreatment of many high-risk patients. As many randomized trials demonstrated a consistent relative risk reduction in adverse CV outcomes per unit reduction in BP [10] and a substantial proportion of excess CV events attributable to BP occur in patients with BP levels below the traditional threshold for hypertension diagnosis [11,12], it became of utmost importance to identify patients with elevated BP and increased CVD risk that can also derive benefit from BP-lowering treatment [13]. The 2024 ESC guidelines recommend that a 10-year risk of ≥10% be considered sufficiently high for CVD events among persons in the elevated BP category.

As for the 2023 ESH guidelines, BP values are classified as optimal (<120/80 mmHg), normal (120–130/80–85 mmHg), high–normal (130–140/85–90 mmHg), grade 1 hypertension (140–160/90–100 mmHg), grade 2 hypertension (160–180/100–110 mmHg), and grade 3 hypertension (>180/110 mmHg). In addition to providing a BP-based classification, the 2023 ESH guidelines are the only guidelines recommending that hypertension also be staged as stage 1 (uncomplicated hypertension), stage 2 (presence of HMOD, diabetes, or CKD stage 3), or stage 3 (presence of CVD or stage 4 or 5 CKD) [1]. By contrast, the 2017 ACC/AHA guidelines classified BP levels as normal (<120/80 mmHg), elevated (systolic BP of 120–129 mmHg), hypertension stage 1 (130–139/80–89 mmHg), and hypertension stage 2 (≥140/90 mmHg) [5], which remained the same in the 2025 ACC/AHA guidelines [2]; the same cutoff was set in most national guidelines of the Asia–Pacific region, e.g., [14].

For the diagnosis of hypertension, an office BP ≥ 140/90 mmHg was set in the most national guidelines of the Asia–Pacific region [14]. As shown in Table 2, 2023 ESH, 2024 ESC, and 2025 ACC/AHA guidelines showed some differences, particularly in the classification of high BP and AH, since American guidelines define a BP > 130–139/80–89 already as the first stage of hypertension, and in BP targets for treatment. In fact, controversies still exist among experts on which would be the most effective target and differences among the three documents may be derived from different years of release, differences in health systems, risk factors, and scores used for risk stratification among the continents.

The current recommendation for hypertensive patients or people with elevated BP who receive treatment is to achieve a target of 120–129/70–79 mmHg, provided treatment is well-tolerated. This recommendation is supported by evidence from RCTs and from meta-analyses of RCTs which established that this treatment target reduces CVD events in adults with hypertension or elevated BP and high or very high CVD risk [4]. Relaxed targets (BP as low as reasonably achievable) are recommended in case of treatment intolerance, adults ≥85 years, symptomatic orthostasis, moderate-to-severe frailty, or limited life expectancy, an important issue by and large disregarded by the 2025 ACC/AHA guidelines. Most countries in the Asia–Pacific region recommend lower BP targets of <130/80 mmHg in specific patient populations (e.g., younger, overweight patients, smokers, and patients with other cardiovascular risk factors, in individuals younger than 75 years or who have established CVD or CKD, diabetes, or use of anti-thrombotic drugs) [14].

The following question is asked: is this target feasible in the clinical practice? The 2024 ESC guidelines propose different approaches to achieve this stricter BP goal, discussing the benefits and limitations of each strategy and particularities in special populations that will be discussed in Section 4. As a matter of fact, the 2025 ACC/AHA refers to implementation strategies based upon team-based care whose feasibility is uncertain. Then, the reality is to face potential comorbidities, polytherapy, and patient compliance to therapy and short follow-up visits.

The first step recommended for both the hypertensive population and the elevated BP population with established CVD, HMOD, diabetes, moderate-to-severe CKD, familial hypercholesterolemia, or high CVD risk is non-pharmacological interventions such as physical activity, increase in potassium intake, reduction in sodium chloride (table salt) intake, optimization of weight management and diet, reduction in alcohol intake, and smoking cessation; the difference is that for the first group, those strategies should be started with pharmacological treatment without delay and for the latter, they may be performed alone as initial intervention, upholding the pharmacological treatment for 3 months after which the patient should be reevaluated and if the BP is still not at the optimal target, initiation of pharmacological therapy is recommended. At variance with these recommendations, the 2023 ESH guidelines uphold a more careful approach to high-risk patients with high–normal BP limiting pharmacological treatment only in the presence of coronary artery disease.

As for pharmacological treatment initiation, international guidelines recommend angiotensin-converting enzyme (ACE) inhibitors, angiotensin receptor blockers (ARBs), dihydropyridine calcium channel blockers (CCBs), and thiazide or thiazide-like diuretics as the first-line BP-lowering agents [4,5]. A relevant difference in the 2024 ESC from the 2023 ESH relates to the downgrading of beta-blockers from first-line antihypertensive medications to drugs to be used in the presence of compelling indications (angina, ischemic heart disease, heart failure with reduced ejection fraction, or for controlling heart rate) because of reduced cerebrovascular protection and tolerability. Following the 2017 edition, the 2025 ACC/AHA guidelines also maintain beta-blockers as secondary agents for BP-lowering therapy, since evidence shows the effect of renin–angiotensin system (RAS) blockers and CCBs on preventing progression of HMOD is superior to beta-blockers. On the other hand, commonly prescribed anti-hypertensives in the Asia–Pacific region include CCBs, RAS blockers, β-blockers, and diuretics [14].

The guidelines recommend initial two-drug therapy, which can be performed as either a separate or fixed-dose combination, with the second one being preferred as it simplifies posology and helps to improve patients’ adherence to treatment. After 1–2 months, if BP is still uncontrolled, low-dose triple combination therapy could be used, and the next step is maximally tolerated triple combination therapy. When therapy and adherence with the above-mentioned drug classes are optimized but insufficient to reach BP goals, other drug classes can be used for treating hypertension, the MRA being the first choice when this next step is needed to reach the BP target. Alpha-blockers, hydralazine (scarcely or not used in most European countries anyway), or a potassium-sparing diuretic should be considered as next step. All of the three analyzed guidelines in this document are in line with the pharmacological therapy strategy reported above and all of them agree on the importance of the non-pharmacological intervention to be performed in parallel with the pharmacological therapy.

This stepwise approach is surely proposed to provide a safe and stable approach to BP, although planning re-evaluation after a few months may often be challenging due to long waiting lists for visits and limited access to the office setting. Therefore, the answer is that this may be feasible if patients are trained for home-monitoring programs and possible re-evaluations with clinicians through teleconsultations are planned.

Renal denervation, a catheter-based procedure that ablates the renal sympathetic nerves, is also recommended in the 2023 ESH and the 2024 ESC guidelines as an adjunctive treatment for resistant hypertension or specific population. According to the guidelines, this procedure may be considered for patients with an eGFR > 40 mL/min/1.73 m^2^ who have uncontrolled BP, despite BP-lowering therapy with >3 agents and with increased CVD risk, after discussing with the patient the risks and potential benefits of the procedure and coming to a shared decision [15]. The 2017 ACC/AHA guidelines do not recommend renal denervation as BP-lowering strategy as its publication was prior to the more recent and new generation trials on renal denervation. As for the 2025 ACC/AHA, renal denervation is considered as an additional option in managing resistant hypertension.

However, most but not all the latest trials on renal denervation demonstrated modest efficacy of this intervention in lowering blood pressure in patients on >3 BP-lowering agents, and individual patient response vary substantially. Another limitation of renal denervation is the cost as most of these procedures are conducted in specialized centers in the context of clinical trials. Although further research is clearly needed, particularly in terms of patient selection and long-term efficacy, renal denervation represents a novel and potential effective treatment strategy for patients with uncontrolled blood pressure or who have multiple medication intolerances [16], increasing the range of treatments available for hypertensive patients and facilitating the achievement of the stricter proposed BP target.

The main differences among the guidelines regarding the classification of HTN, treatment target, and indication for first-line BP-lowering agents are listed in Table 2.

## 4. Special Populations

The new guidelines also discuss the management of hypertension in special populations such as patients with diabetes mellitus, CKD, pregnancy, elderly, and resistant hypertension. In these patients, particular attention should be paid to the choice of the specific drug.

### 4.1. Diabetes Mellitus

Patients with diabetes mellitus often present elevated BP or HTN and are about twice as likely to suffer a major CVD event compared with those without diabetes [17]. Even though, on average, a patient with diabetes is already at high risk for CVD outcome, the 2024 ESC guideline recommends using the SCORE2-Diabetes, to better estimate the individual CVD risk in patients with type 2 diabetes mellitus and aged < 60 years. The 2025 AHA/ACC guidelines consider patients with HTN and concomitant diabetes to be at high CVD risk (PREVENT ≥ 7.5%) and the 2023 ESH guidelines consider patients with diabetes to be classified at moderate CVD risk only when with a well-controlled, short-standing duration of the disease (less than 10 years) with no evidence of HMOD and no additional CV risk factors. The BP target in this population is the same as for the general population of 120–129/70–79 mmHg, if feasible and tolerated, according to the 2024 ESC guidelines. For the 2025 AHA/ACC guidelines, the treatment goal is <130 mmHg, with encouragement to achieve an SBP < 120 mmHg to reduce CVD morbidity and mortality, while the 2023 ESH guidelines recommend the target for treatment in this population to reach an SBP < 130 mmHg and DBP < 80 mmHg but not below SBP 120 mmHg and DPB 70 mmHg (Table 1).

As is known, albuminuria is more common in diabetes and, for this reason, ACE inhibitors and ARBs are the preferred BP-lowering agents in this population [18].

### 4.2. Chronic Kidney Disease

The pathogenesis of HTN and CKD are closely bonded and patients with CKD present CVD as one of the largest contributors to mortality, with hypertension being a major risk factor. For this fact, the 2024 ESC and 2023 ESH agree that adults with moderate-to-severe CKD (persons with eGFR of <60 mL/min/1.73 m^2^ and/or albuminuria of ≥30 mg/g) and elevated BP are at sufficiently high risk to be considered for BP-lowering therapy. Reducing BP in patients with CKD not only reduces CVD events and mortality but also reduces the progression of CKD and the incidence of end-stage renal disease [19]. The systolic BP target for adults with CKD who have eGFR > 30 mL/min/1.73 m^2^ is of 120–129 mmHg, for those with lower eGFR or renal transplantation the guidelines recommend an individualized BP target. Both ACE inhibitors and ARBs reduce the risk of CVD events and kidney failure in patients with CKD; however, ACE inhibitors appear to be superior to ARBs for that purpose [4]. Patients with CKD usually require combination therapy, and this should be initiated as a combination of an RAS inhibitor and a CCB or diuretic.

As a great novelty of the latest years, the 2024 ESC and 2023 ESH guidelines also recommend the use of sodium–glucose co-transporter 2 (SGLT2) inhibitors in patients with CKD and eGFR > 20 mL/min/1.73 m^2^ to improve outcomes in the context of their modest BP-lowering properties, though these drugs are not currently marketed for BP-lowering effects alone.

### 4.3. Pregnancy

Hypertension in pregnancy is defined as systolic BP of ≥140 mmHg and/or diastolic BP of ≥90 mmHg. Its prompt diagnosis is crucial as it represents the second leading cause of maternal death after maternal peri-partum hemorrhage and women that present hypertensive disorders during pregnancy are at greater risk of developing hypertension and presenting CVD events later in life [20]. Hypertensive disorders in pregnancy includes chronic hypertension (precedes pregnancy, develops before 20 weeks of gestation, or persists for >6 weeks post-partum), gestational hypertension (develops after 20 weeks of gestation and usually resolves within 6 weeks post-partum) and pre-eclampsia (gestational hypertension accompanied by new onset proteinuria and other organ dysfunction, including acute kidney injury, liver dysfunction, neurological complications, or hematological complications).

There is a lack of evidence that support a BP target as low as 120–129/70–79 mmHg and, to this day, the recommendation is to lower BP to a systolic BP of <140 mmHg and diastolic BP of 80–90 mmHg in pregnant women [4].

The BP-lowering agents recommended in pregnancy are beta-blockers (though atenolol should be avoided as it is correlated with fetal growth restriction), dihydropyridine CCBs (nifedipine is usually considered first choice), and methyldopa. Hydralazine can be particularly effective for severe hypertension, which is defined as a BP of ≥160/90 mmHg, and can be administered intravenously. As for RAS inhibitors, they are not recommended in pregnancy due to adverse fetal and neonatal outcomes.

Pre-eclampsia is cured by delivery, which should be performed at 37 weeks of pregnancy, or earlier in high-risk cases. Women with moderate-to-high risk of pre-eclampsia should be advised to take 100–150 mg of aspirin daily from gestational weeks 12–36 [4].

### 4.4. Elderly

The maintenance of the same targets for younger and elderly patients is one of the most discussed questions regarding BP control. The current available evidence from RCTs do not deny neither confirm that very old patients with hypertension may benefit from BP-lowering treatment down to a target of 120–129/70–79 mmHg. The 2024 ESC guidelines recommend the same target of BP among older patients aged < 85 years as for younger people, provided that BP-lowering treatment is well-tolerated. However, the 2024 ESC guidelines recommend a more personalized approach to older patients (≥85 years) according to their frailty status and presence or not of orthostatic hypotension. Concerning the elderly, not only the management of BP is considered essential for improving quality of life and outcomes but also addressing whether reversible causes of frailty are present and can be managed by, for example, treating underlying comorbidities or undergoing supervised muscle-strengthening physiotherapy or supervised exercise and co-ordination and balance training [21], is of utter importance. Moreover, BP control is usually important in elderly patients with comorbidities not only to reduce AH as a cardiovascular risk factor, but also to reduce the risk of complications derived by potential comorbidities such as aortic aneurysms and cerebrovascular disease. This should encourage clinicians to maintain the same approach in these patients, parallel to caution while choosing the drugs, considering potential effects on renal function, electrolyte balance, and potential interactions with other drugs.

### 4.5. Resistant Hypertension

Resistant hypertension is defined as a BP value remaining above target despite ≥3 BP-lowering agents of different classes at maximally tolerated doses, of which one is a diuretic [22]. In patients with eGFR < 30 mL/min/1.73 m^2^, an adequately up-titrated loop diuretic is necessary to define resistant hypertension [4]. First of all, the 2024 ESC guidelines recommend that non-adherence to treatment and secondary hypertension should be investigated in these patients, since these may represent a higher percentage of masked resistant hypertension. Moreover, this suggests that these patients should be referred to specialized centers. Most patients with resistant hypertension will require the addition of a non-first-line BP-lowering agent and the 2024 ESC, the 2023 ESH, and the 2017 ACC/AHA guidelines all favor spironolactone, a mineralocorticoid receptor antagonist (MRA), as the first-line agent for resistant hypertension; however, is important to highlight that the efficacy and safety of spironolactone in patients with significant renal impairment have not yet been established, so its use should be restricted to patients with an eGFR > 30 mL/min/1.73 m^2^, with caution for the risk of hyperkalemia, especially when associated with RAS inhibitors.

In unresponsive patients, renal denervation, as discussed above, is one of the therapeutic options to consider.

## 5. Conclusions

The latest guidelines on arterial hypertension elaborated from different societies show considerable differences, but the overall message remains similar: to implement screening modalities and give more emphasis for out-of-office measurements, initiate pharmacological therapy in lower BP ranges according to the CVD risk stratification, and to reach lower targets of BP for reducing CVD events.

Considering a wider range of strategies from screening/diagnosis to treatment options, the physicians have more capability to overgo inertia and to improve patient’s adherence and compliance to treatment, becoming positively feasible to reach the new, stricter, goal of BP recommended in the different guidelines. The final aim is to reduce CVD burden at the individual level and societal level by improving patients’ life expectancy and quality of life on one hand, and reducing costs for health systems, reducing hospitalizations, and lowering expenses on emergency and intensive care on the other hand, finally preventing productivity losses due to premature death or disability [3].

## Figures and Tables

**Table 1:** Revised recommendations for ESC guidelines and ACC/AHA guidelines. For the sake of clarity and editorial space, we did not include the 2023 ESH guidelines because of their substantial homology with the 2018 ESC/ESH guidelines. ABPM, ambulatory blood pressure monitoring; ACE, angiotensin-converting enzyme; ARB, angiotensin receptor blocker; ASCVD, atherosclerotic cardiovascular disease; BP, blood pressure; CCB, calcium channel blocker; CKD, chronic kidney disease; CV, cardiovascular; CVD, cardiovascular disease; dBP, diastolic blood pressure; HBPM, home blood pressure monitoring; LoE, level of evidence; N/A, not applicable; RDN, renal denervation; sBP, systolic blood pressure.

2018 ESC/ESH	2024 ESC	2017 ACC/AHA	2025 ACC/AHA
**Definition and classification of elevated blood pressure and hypertension**			
BP should be classified as optimal, **normal, high–normal, or grades 1–3 hypertension**, according to office BP.	BP should be categorized as **non-elevated BP, elevated BP, and hypertension** to aid treatment decisions.	BP should be categorized as **normal, elevated, or stage 1 or 2** hypertension to prevent and treat high BP.	In adults, BP should be categorized as **normal**, **elevated, or stage 1 or 2 hypertension** to prevent and treat high BP.
CV risk assessment with SCORE system **is recommended** for hypertensive patients who are not already at high or very high risk.	CV risk assessment with SCORE 2 is **recommended** among individuals aged 40–69 years with elevated BP who are not already considered at increased risk. **SCORE2-OP is recommended** for assessing the 10-year risk of fatal and non-fatal CVD among individuals aged ≥ 70 years with elevated BP who are not already considered at increased risk.	Use of BP-lowering medications **is recommended** in patients with prior CVD and an average of SBP ≥ 130 mmHg or an average DBP ≥ 80 mmHg and for primary prevention in adults with an estimated 10-year ASCVD risk of ≥10% and an average sBP ≥ 130 mmHg or an average dBP ≥ 80 mmHg and with an estimated 10-year ASCVD risk < 10% and an sBP ≥ 140 mmHg or a dBP ≥ 90 mmHg.	In adults with hypertension without clinical CVD but with diabetes or CKD or at increased 10-year CVD risk (i.e., ≥7.5% based on **PREVENT**), initiation of BP-lowering medications **is recommended** when average SBP is ≥130 mmHg and average DBP is ≥80 mmHg or with 10-year CVD risk < 7.5% based on PREVENT if average sBP remains ≥ 130 mmHg or average dBP remains ≥ 80 mmHg after a 3- to 6-month trial of lifestyle intervention.
**Diagnosing hypertension and investigating underlying causes**			
Screening: Every 5 years if BP remains optimal; Every 3 years if BP remains normal; If BP remains high–normal, further BP recording, at least annually; In older patients (>50 years), more frequent screening of office BP should be considered. Diagnosis: repeated office BP measurements > one visit, except when hypertension is severe or out-of-office BP measurement with ABPM and/or HBPM	Opportunistic screening **should be considered**: At least every 3 years for adults aged < 40 years; At least annually for adults aged ≥ 40 years. In individuals with elevated BP and without treatment, repeat BP measurement and risk assessment within 1 year. Diagnosis: ⇒ Screening office BP = 140–159/90–99 mmHg, the diagnosis **is recommended** to be based on out-of-office BP measurement with ABPM and/or HBPM or at least made on repeated office BP measurements on > one visit. ⇒ BP 160–179/100–109 mmHg has to be confirmed as soon as possible (e.g., within 1 month) by either home or ambulatory BP. ⇒ BP ≥180/110 mmHg hypertensive emergency should be excluded.	Proper methods are **recommended** for accurate measurement and documentation of BP. Out-of-office BP measurements are recommended to confirm the diagnosis of hypertension and for titration of BP-lowering medication. In adults being treated for hypertension with elevated HBPM readings suggestive of masked uncontrolled hypertension, confirmation by ABPM **might be reasonable** before intensification of antihypertensive treatment.	Standardized methods are **recommended** for the accurate measurement and documentation of in-office BP. In adults, **it is** **reasonable** to use the oscillometric method with an automated device over the auscultatory method. In suspected hypertension, out-of-office BP measurements by either ABPM or HBPM are recommended to confirm the diagnosis of hypertension. In adults being treated for hypertension, HBPM **is recommended** for monitoring the titration of BP-lowering medication, along with cointerventions such as patient education, telehealth counseling, and clinical interventions.
**Blood pressure targets**			
First objective of treatment: Lower BP to <140/90 mmHg in all patients and target to <130/80 mmHg in most patients. A dBP target of <80 mmHg **should be considered** for all hypertensive patients. In older patients (aged ≥ 65 years) receiving BP-lowering drugs sBP should be targeted to 130–139 mmHg.	Treated systolic BP values in most adults should be targeted to **120–129** mmHg. If on-treatment sBP is 120–129 mmHg but **dBP is ≥ 80** mmHg, intensifying BP-lowering treatment to achieve an on-treatment diastolic BP of 70–79 mmHg **may be considered**. **Personalized** and more lenient sBP targets (e.g., <140 mmHg) should be considered in the following cases: Pre-treatment, symptomatic, orthostatic hypotension; Age ≥85 years; Moderate-to-severe frailty at any age; Limited predicted lifespan (<3 years).	BP-lowering medications are **recommended** for the following: - Secondary prevention in patients with clinical CVD and an average SBP ≥ 130 mmHg (**LoE A**) or an average DBP ≥ 80 mm Hg (**LoE C**); - Primary prevention with estimated 10-year ASCVD risk ≥ 10% and an average SBP ≥ 130 mm Hg or an average DBP ≥ 80 mmHg; - Primary prevention with no history of CVD and with an estimated 10-year ASCVD risk < 10% and an SBP of ≥140 mm Hg or a DBP ≥ 90 mmHg. If confirmed hypertension and known CVD or 10-year ASCVD risk ≥ 10% or higher, a BP target < 130/80 mmHg **is recommended.** If confirmed hypertension, without additional markers of increased CVD risk, a BP target < 130/80 mmHg **may be reasonable**.	BP-lowering medications is recommended in the following cases: - Average SBP is ≥140 mm Hg (**LoE A**); - Average DBP is ≥90 mm (**LoE A**). In adults with confirmed hypertension at increased risk for CVD, an SBP goal of at least <130 mm Hg, with encouragement to achieve SBP < 120 mm Hg, **is recommended** to reduce the risk of cardiovascular events and total mortality. In adults with confirmed hypertension who are not at increased risk for CVD, an SBP goal of <130 mmHg, with encouragement to achieve SBP < 120 mm Hg, **may be reasonable** to reduce risk of further elevation of BP.
**Treatment**			
ACE inhibitors, ARBs, beta-blockers, CCBs, and diuretics (thiazides and thiazide-like drugs) are indicated. If BP is not controlled with a three-drug combination (resistant hypertension), spironolactone is recommended or, if not tolerated, other diuretics such as amiloride or higher doses of other diuretics, a beta-blocker, or an alpha-blocker.	Among all BP-lowering drugs, ACE inhibitors, ARBs, dihydropyridine CCBs, and diuretics (thiazides and thiazide-like drugs) are **recommended** as first-line treatments to lower BP. Beta-blockers should be reserved for adults with compelling indications because of a lower effectiveness in preventing strokes and a less favorable side effect profile. If BP is not controlled with a three-drug combination (resistant hypertension) and if spironolactone is not effective or tolerated, **consider** (1) eplerenone instead of spironolactone, or a beta-blocker and, (2) a centrally acting BP-lowering medication, an alpha-blocker, hydralazine, or a potassium-sparing diuretic.	For initiation, first-line agents include thiazide diuretics, CCBs, and ACE inhibitors or ARBs. Initiation of antihypertensive drug therapy with two first-line agents of different classes is **recommended** in adults with stage 2 hypertension and an average BP > 20/10 mmHg their BP target. Initiation with a single antihypertensive drug **is reasonable** in adults with stage 1 hypertension and BP goal < 130/80 mm Hg with dosage titration and sequential addition of other agents to achieve the BP target.	For initiation, thiazide-type diuretics, long-acting dihydropyridine CCB, and ACEi or ARB **are recommended** as first-line therapy to prevent CVD (I). In adults with stage 2 hypertension (SBP ≥ 140 mm Hg and DBP ≥ 90 mm Hg), initiation with 2 first-line agents of different classes, ideally in a single-pill combination is **recommended**. In adults with stage 1 hypertension (SBP 130–139 mmHg and DBP 80–89 mmHg), initiation with a single first-line drug **is reasonable**. In adults with hypertension, simultaneous use of an ACEi, ARB, and/or renin inhibitor in combination is **not recommended** due to the potential for harm.
Use of device-based therapies **is not recommended** for the routine treatment of hypertension, unless in the context of clinical studies and RCTs, until further evidence regarding their safety and efficacy becomes available.	If performed at a medium-to-high volume center, catheter-based renal denervation (RDN) **may be considered** for resistant hypertension patients who have uncontrolled BP despite a three BP-lowering drug combination (including a thiazide or thiazide-like diuretic), and who express a preference to undergo renal denervation.	N/A	All patients with hypertension who are being considered for RDN should be evaluated by a multidisciplinary team with specific expertise.
**Managing special populations**			
Diabetes mellitus			
Antihypertensive drug treatment **is recommended** for people with diabetes when office BP is ≥140/90 mmHg.	In most adults with elevated BP and diabetes, after a maximum of 3 months of lifestyle intervention, BP-lowering treatment **is recommended** for those with **office BP ≥ 130/80** mmHg. In persons with diabetes who are receiving BP-lowering drugs, it is recommended to target systolic BP to 120–129 mmHg, if tolerated.	In adults with diabetes and hypertension, ACEi or ARB **may be considered** in the presence of albuminuria.	In adults with diabetes and hypertension, ACEi or ARB **are recommended** in the presence of CKD as identified by eGFR < 60 mL/min/1.73 m^2^ or albuminuria ≥ 30 mg/g and should be considered with mild albuminuria (<30 mg/g).
Chronic kidney disease			
In patients with CKD, an office **BP ≥ 140/90** mmHg **is recommended** with lifestyle advice and BP-lowering medication.	In patients with moderate-to-severe CKD and confirmed **BP ≥ 130/80** mmHg, lifestyle optimization and BP-lowering medication **are recommended** to reduce CVD risk.	In adults with hypertension and CKD (stage ≥ 3 or stage 1 and 2 with albuminuria ≥ 300 mg/d, or ≥300 mg/g A/C ratio or the equivalent in the first morning void), treatment with an **ACEi is reasonable** and treatment with an ARB **may be reasonable** if an ACEi is not tolerated.	For adults with hypertension and CKD with eGFR < 60 mL/min/1.73 m^2^ with albuminuria of ≥30 mg/g, **RAASi** (either with ACEi or ARB but not both) **are recommended** to decrease CVD and delay progression of kidney disease.
**It is recommended** to lower sBP to a range of 130–139 mmHg.	In treated adults with moderate-to-severe CKD with eGFR > 30 mL/min/1.73 m^2^, **it is recommended** to target systolic BP to 120–129 mmHg, if tolerated. Individualized targets should be used for eGFR < 30 or kidney transplantation.		
Pregnancy			
SBP ≥ 170 mmHg or dBP ≥ 110 mmHg in a pregnant woman is an emergency, and admission to hospital **is recommended**.	Systolic BP **≥ 160** mmHg or diastolic BP ≥ 110 mmHg in pregnancy can indicate an emergency, and immediate hospitalization **should be considered.**	N/A	Pregnant individuals with sBP ≥ **160 mmHg** or **dBP ≥ 110** mmHg confirmed on repeat measurement within 15 min **should receive** antihypertensive medication to lower BP within 30 to 60 min.
Resistant hypertension			
Only pharmacological treatment recommended.	Catheter-based renal denervation **may be considered** for resistant hypertension patients after a shared risk–benefit discussion and multidisciplinary assessment.	N/A	In adults with resistant hypertension, screening for primary aldosteronism **is recommended**.

**Table 2:** Main differences between 2024 ESC, 2023 ESH, and 2025 AHA/ACC arterial hypertension guidelines. ACE, angiotensin-converting enzyme; ARB, angiotensin receptor blocker; BP, blood pressure; CCB, calcium channel blocker; CKD, chronic kidney disease; HMOD, hypertension-mediated organ damage; HTN, hypertension; sBP, systolic blood pressure.

	2024 ESC Guidelines	2023 ESH Guidelines	2025 AHA/ACC Guidelines
Classification of Hypertension	Non-elevated BP: <120/70 mmHg	Optimal BP <120/80 mmHg	Normal BP: <120/80 mmHg
	Elevated BP: 120–139/70–89 mmHg	Normal BP: 120–130/80–85 mmHg	Elevated BP: 120–129 mmHg
		High–normal: 130–140/85–90 mmHg	HTN stage 1: 130–139/80–89 mmHg
	Hypertension: >140/90 mmHg	Grade 1 HTN: 140–160/90–100 mmHg	HTN stage 2: ≥140/90 mmHg
		Grade 2 HTN: 160–180/100–110 mmHg	
		Grade 3 HTN: >180/110 mmHg	
Threshold for pharmacological treatment initiation	BP ≥ 140/90 mmHg irrespective of age	sBP ≥ 160 mmHg if age ≥ 80 y	BP ≥ 140/90 mmHg irrespective of age
	BP 130–139/80–89 mmHg despite 3 months of lifestyle intervention if established CVD, HMOD, diabetes, moderate-to-severe CKD, familial hypercholesterolemia, or high CVD risk (10-y SCORE2/SCORE2-OP > 10%)	BP ≥ 140/90 mmHg if age 18–79 y	BP ≥ 130/80 mmHg if established CVD, diabetes, CKD, or the patient is at increased short-term risk of CVD (10-y PREVENT ≥ 7.5%)
		BP ≥ 130/80 mmHg if established CVD, predominantly CAD	BP ≥ 130/80 mmHg and patients are not at increased risk of CVD after 3–6 months of lifestyle intervention
Treatment target	120–129/70–79 mmHg	First objective <140/90 mmHg in all patients. If tolerated, BP values should be targeted to 130/80 mmHg or lower, to personalize in some clinical conditions (e.g., CKD, >65 y).	<130/80 mmHg
	Relaxed targets in case of treatment intolerance, >85 years old, symptomatic orthostasis, moderate-to-severe frailty, or limited expectancy of life.	Never target BP values to <120/70 mmHg because of the lack of consistent evidence that this has a protective effect.	No mention of more relaxed targets.
**First-line BP-lowering agents**	ACE inhibitors, ARBs, dihydropyridine CCBs, and thiazide or thiazide-like diuretics.	ACE inhibitors, ARBs, dihydropyridine CCBs, thiazide or thiazide-like diuretics, and beta-blockers.	ACE inhibitors, ARBs, dihydropyridine CCBs, and thiazide or thiazide-like diuretics.
	Beta-blockers are recommended as combination therapy with one of the major classes above when there is a compelling indication (e.g., angina).		Beta-blockers are recommended as combination therapy with one of the major classes above when there is a compelling indication (e.g., angina).

## Data Availability

No new data were created or analyzed in this study. Data sharing is not applicable to this article.
